# *Demodex gatoi *-associated contagious pruritic dermatosis in cats - a report from six households in Finland

**DOI:** 10.1186/1751-0147-51-40

**Published:** 2009-10-20

**Authors:** Seppo AM Saari, Kirsi H Juuti, Joanna H Palojärvi, Kirsi M Väisänen, Riitta-Liisa Rajaniemi, Leena E Saijonmaa-Koulumies

**Affiliations:** 1 Department of Basic Veterinary Sciences (FINPAR), Faculty of Veterinary Medicine, University of Helsinki, Finland; 2 CatVet Cat Clinic, Helsinki, Finland; 3 Espoo Animal Hospital, Espoo, Finland; 4 Private Veterinary Clinic, Outokumpu, Finland; 5 Lemmikki Veterinary Clinic, Oulu, Finland; 6 Department of Equine and Small Animal Medicine, Faculty of Veterinary Medicine, University of Helsinki, Finland

## Abstract

**Background:**

*Demodex gatoi *is unique among demodectic mites. It possesses a distinct stubby appearance, and, instead of residing in the hair follicles, it dwells in the keratin layer of the epidermis, causing a pruritic and contagious skin disease in cats. Little is known of the occurrence of *D. gatoi *in Europe or control of *D. gatoi *infestation.

**Case presentation:**

We describe *D. gatoi *in 10 cats, including five Cornish Rex, two Burmese, one Exotic, one Persian and one Siamese, living in six multi-cat households in different locations in Finland containing 21 cats in total. Intense pruritus was the main clinical sign. Scaling, broken hairs, alopecia and self-inflicted excoriations were also observed.

Diagnosis was based on finding typical short-bodied demodectic mites in skin scrapings, skin biopsies or on tape strips. Other pruritic skin diseases, such as allergies and dermatophytoses, were ruled out. In one household, despite finding several mites on one cat, all six cats of the household remained symptomless.

Amitraz used weekly at a concentration of 125-250 ppm for 2-3 months, proved successful in three households, 2% lime sulphur weekly dips applied for six weeks in one household and peroral ivermectin (1 mg every other day for 10 weeks) in one household. Previous trials in four households with imidacloprid-moxidectin, selamectin or injected ivermectin given once or twice a month appeared ineffective.

**Conclusion:**

*D. gatoi*-associated dermatitis is an emerging contagious skin disease in cats in Finland. Although pruritus is common, some cats may harbour the mites without clinical signs. In addition, due to translucency of the mites and fastidious feline grooming habits, the diagnosis may be challenging. An effective and convenient way to treat *D. gatoi *infestations has yet to emerge.

## Background

The first report of *Demodex gatoi *(Desch & Steward 1999) in cats was in 1981 [1]. *D. gatoi *is a unique demodectic mite in many respects. Instead of residing in the hair follicles or sebaceous glands, it dwells in the superficial keratin layer (stratum corneum) of the skin. It differs from other demodectic mites also by being a primarily pathogenic parasite, causing a skin disease that tends to be contagious and pruritic [2-4].

Earlier reports describe the feline demodecosis caused by *D. gatoi *as a rare skin disease more often seen in localized enzootic regions in the southern and south-eastern parts of the United States [5]. However, some recent books and articles have highlighted its increasing significance, and nowadays *D. gatoi *is considered "an important differential diagnosis for a pruritic cat, emerging as a common cause of pruritic skin disease in the Southern United States"[6]. In Europe, information on the occurrence of *D. gatoi *is sparse. In France, the mite was reported in two Bengal cats [7] and later in three other cats [8]. It has also been diagnosed in the UK [9]. However, despite its increasing importance, little is known of the biology of the mite or the mode of transmission, pathogenesis or treatment options of the *D. gatoi*-associated dermatosis.

Here, we describe the clinical features and problems associated with the diagnostics and treatment of *D. gatoi *infestation in 10 cats from six households in Finland.

## Case presentations

**Figure 1:**
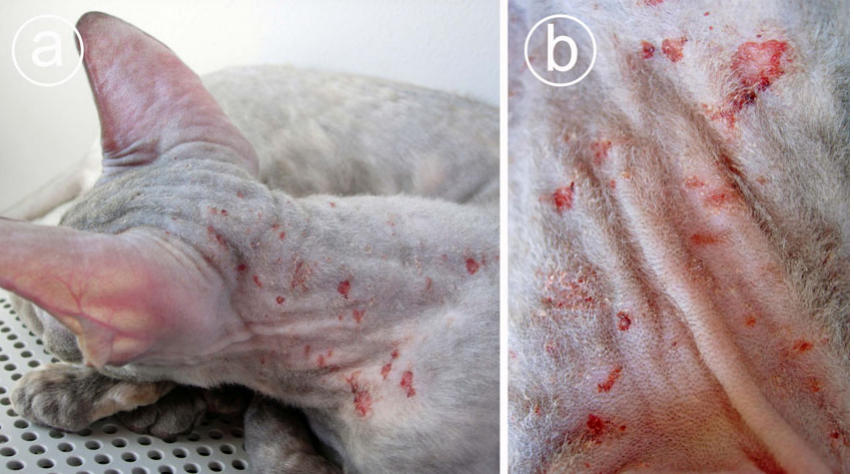
**A Cornish Rex cat with *Demodex gatoi-*associated pruritic dermatosis**. In Figure 1a, Alopecia, mild crusting and severe self-inflicted excoriations and wounds are visible on the skin of the head and neck, Case 4A. Figure 1b is a close-up photograph depicting the skin lesions on the neck.

**Figure 2:**
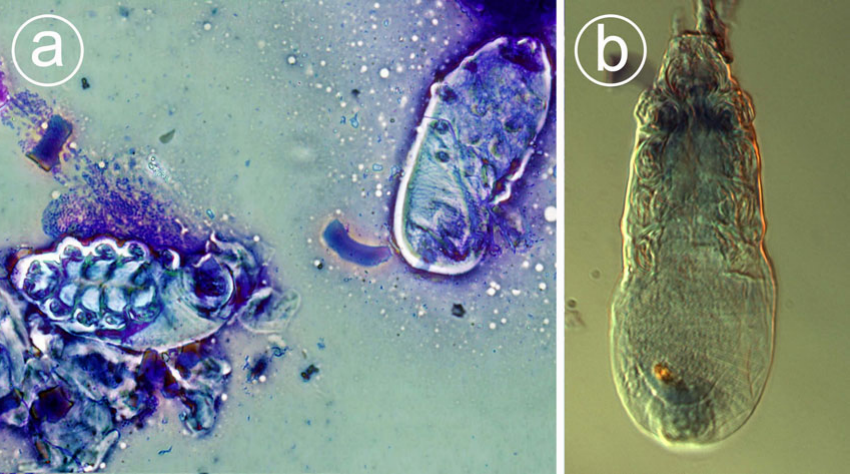
***Demodex gatoi *mites as seen in a skin scraping under a light microscope**. Figure 2a is a May-Grünwald-Giemsa-stained skin scraping showing an adult mite (on the left) and a nymphal stage (on the right). The flaky bluish material around the mites is keratin. 2b depicts unstained adult *D. gatoi *under a microscope equipped with differential interference contrast (DIC). The average length of a *D. gatoi *mite is 100 μm.

**Figure 3:**
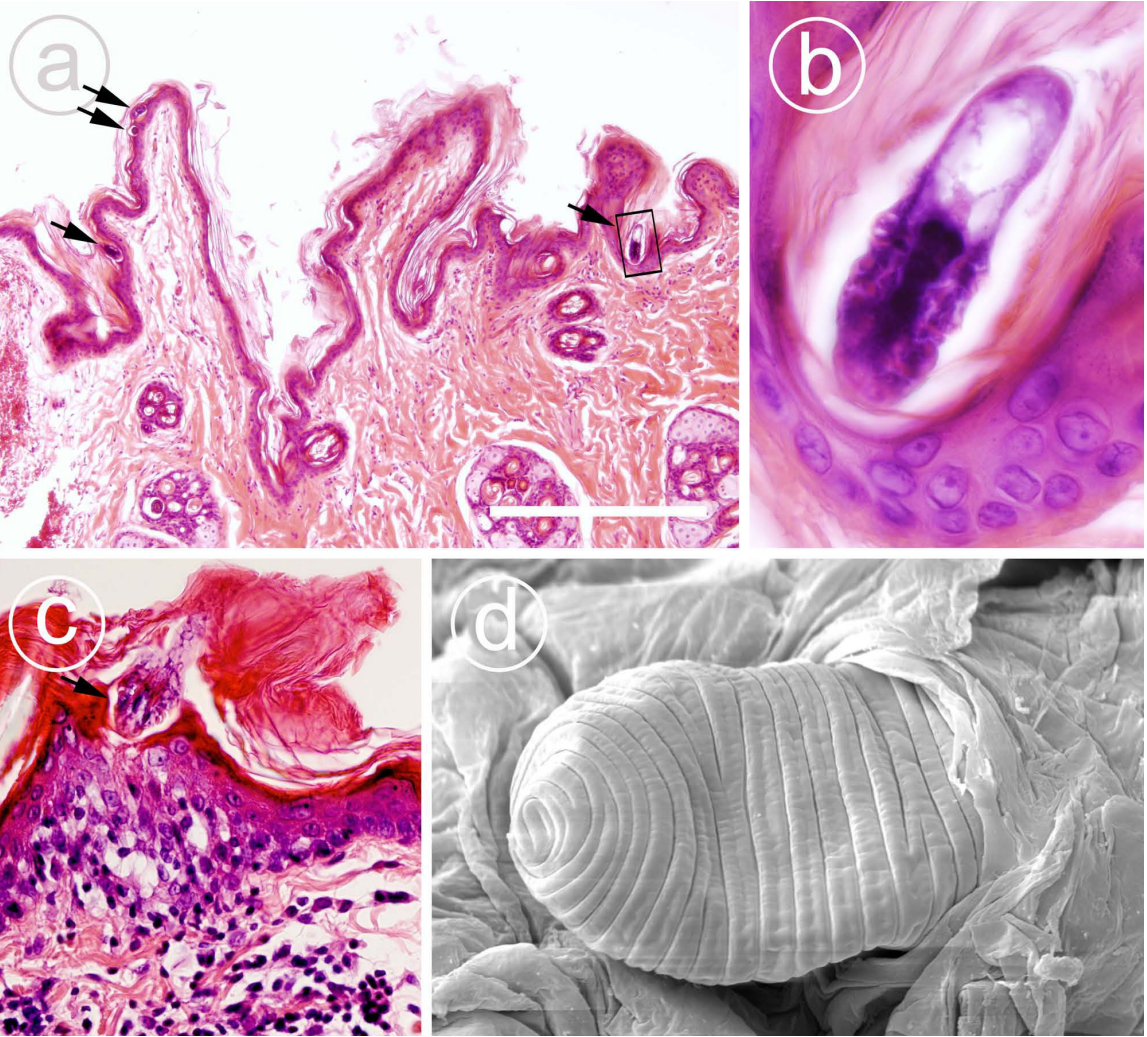
**Histopathology of feline demodecosis caused by *D. gatoi *and a *D. gatoi *infestation as observed under SEM**. In Figure 3a, numerous *D. gatoi *mites (arrows) can be observed within the superficial keratin layer of the epidermis. The area in the rectangle is enlarged in Figure 3b, Case 1C, H&E, scale bar = 500 μm. 3b is a close-up micrograph depicting a *D. gatoi *in the epidermal pit. Figure 3c shows mild to moderate lympho-histiocytic perivascular inflammation in the upper dermis and lymphocytic exocytosis within a mildly acanthotic epidermis. A *D. gatoi *mite (arrow) can be observed within the mildly hyperkeratotic superficial keratin layer, Case 5B, H & E. In SEM micrograph 4d, *D. gatoi *can be seen on the skin surface buried within the superficial keratin layer. The posterior part of the mite (opisthosoma) is visible.

The main historical, clinical and diagnostic findings are summarized in Table 1, and the treatment protocols tested are displayed in Table 2. The clinical cases, morphology of the mites detected and histopathological findings are depicted in the figures as follows: A typical clinical case is depicted in Figure 1, and the morphology of *D. gatoi *mite as seen under a light microscope is shown in Figure 2. Typical histopathology observed in *D. gatoi*-associated dermatosis of the cases presented here is shown in Figure 3. Figure 3d is a scanning electron micrograph showing a *D. gatoi *mite that had partially penetrated the superficial keratin layer of feline skin.

**Table 1:** Signalment, age of onset, duration of symptoms before diagnosis, main clinical signs and findings, presence of *Demodex *mites in skin scrapings, tape strips and biopsies, and results of dermatophyte culture, FeLV and FIV tests in 10 cats with *Demodex gatoi *infestation from six households.

**Case** **n = 10**	**Age**	**Sex**	**Breed**	**Duration of disease before diagnosis**	**Pruritus**	**Alopecia**	**Scaling/crusting**	**Mites in skin scraping/tape strip**	**Mites in skin biopsy**	**Dermatophyte culture**	**FeLV**	**FIV**
**1A**	2 y	F	CRX	4 m	+	+	+	+	+	-	-	-

**1B**	5 y	MN	CRX	6 m	+	+	+	-	-	-	NA	NA

**1C**	5 y	MN	CRX	5 m	+	+	+	+	+	-	NA	NA

**2A**	5 y	F	BUR	7 m	+	+	+	-	+	-	-	-

**3A**	2 y	M	EXO	5 m	+	+	+	+	NA	NA	-	-

**3B**	3 y	MN	PER	4 m	+	+	+	+	NA	NA	-	-

**4A**	6 m	F	CRX	2 m	+	+	+	+	NA	NA		

**5A**	4 m	F	SIA	4 m	+	+	+	-	-	-	NA	NA

**5B**	11 y	FS	BUR	3 m	+	+	+	-	+	-	NA	NA

**6A**	10 m	F	CRX	0 m	-	-	-	+	NA	NA	-	-

F = intact female, MN = neutered male, M = intact male, FS = spayed female, CRX = Cornish Rex, BUR = Burmese, EXO = Exotic, PER = Persian, SIA = Siamese, NA = not available

**Table 2:** Summary of treatments of *Demodex gatoi *infestations evaluated in the present case series consisting of 10 cats from six households. The design of the table is adapted from Mueller [20].

**No. of household** **n = 6 (affected cats + unaffected cats)**	**Cats included** **n = 11**	**Drug and dose evaluated**	**Evaluation criteria**	**Underlying disease identified**	**Outcome**	**Follow-up (months)**
**1** **(3+1)**	1A, IB, 1C	Selamectin^a ^6-12 mg/kg every 30 days for 2 months	SS, CE, SB	Allergy (1C)	UE	0
	
	1A, IB, 1C	Selamectin 6-12 mg/kg every 14 days for 6 months	SS, CE, SB	Allergy (1C)	UE	0
	
	1A, IB, 1C	Amitraz^b ^0.0125% solution dips every 7 days for 12 weeks	SS, CE, SB	Allergy (1C)	E	12

**2** **(1+4)**	2A	Selamectin every 30 days for 5 months	CE, SB	None	E	6

**3** **(2+0)**	3A, 3B	Ivermectin^c ^300 μg/kg SC every 14 days (3 times), followed by selamectin 6-12 mg/kg every 14 days (3 times)	SS, CE	None	UE	0
	
	3A, 3B	Amitraz 0.0125% solution dips every 7 days for 12 weeks	SS, CE	None	E	24

**4** **(1+1)**	4A	Amitraz 0.0125% solution dips every 7 days for 12 weeks	SS, CE	None	E	12

**5** **(2+0)**	5A, 5B	Selamectin 6-12 mg/kg (once), followed by imidacloprid+moxidectin^d ^a month later (once)	CE	None	UE	0
	
	5A, 5B	Ivermectin 1 mg/kg bw PO every 2 days for 10 weeks	CE, SS, SB	None	E	6

**6** **(1+5)**	6A	Lime sulphur dips 2% weekly for 6 weeks	SS	None	E	6

^a^Stronghold^® ^Pfizer Animal Health, bEctodex^®^, Intervet/Schering-Plough Animal Health, cIvomec^®^, Merial, dAdvocate^® ^Bayer Animal Health. SC = subcutaneously, PO = perorally, SS = skin scraping or tape strip, CE = clinical evaluation, SB = skin biopsy, E = effective, UE = uneffective

Household 1 consisted of four Cornish Rex cats. Intensively pruritic dermatosis was observed in three of the cats (Cases 1A, 1B, 1C) in July 2002. Between July and October 2002, these cats had been presented to veterinarians several times. The cats had been treated with corticosteroids and antifungal therapy and fed hypoallergenic food. The diagnosis was made in October 2002 when stubby demodectic mites identified as *D. gatoi *were found in tape strip samples obtained from cats 1A and 1C. Treatment with selamectin was initiated once a month (Table 2), and prednisolone was prescribed to control the intense pruritus. Two months later, multiple skin biopsies were taken from all three pruritic cats and submitted to a histopathological laboratory (Patovet, Helsinki, Finland). In all biopsies, mild hyperplastic perivascular dermatitis with mild hyperkeratosis was detected. Numerous *D. gatoi *mites were observed within the superficial keratin layers in cats 1A and 1C. Treatment was continued by giving selamectin every two weeks for six months. During the treatment pruritus was less intense. However, six months later, the cats were still pruritic and, as numerous mites could still be found in one cat (1C), amitraz bathing with 0.0125% solution was initiated. Within a few weeks, the cats became less pruritic and regrowth of the fur occurred in all cats. Three months later, the fur had completely regrown in all cats, pruritus was absent and no mites were present on tape strips. As these cats had attended several cat shows, both in Finland and abroad, the owner suspected that the source of the *Demodex *infestation was a cat show. A young female Cornish Rex, the youngest cat in the household, had neither symptoms nor mites, but also participated in the treatment protocols.

Household 2 consisted of five Burmese cats. In April 2003, one of the cats (Case 2A) was presented to a small animal practitioner because of intensive pruritus and patchy alopecia. During the next months the cat visited the veterinarian several times and was treated for suspected allergy or dermatophytosis. Six months later, skin biopsies were taken and submitted to a histopathological laboratory (Patovet). In the biopsies, *D. gatoi *mites were observed within the keratin layer of the skin. Mild hyperplastic perivascular dermatitis was also present. All cats were treated with selamectin once a month for five months (Table 2). After the treatment, pruritus had disappeared and fur had regrown on the alopecic sites. Skin scrapings and biopsies were negative for mites. Afterwards, the same cat has had two further pruritic episodes. No mites were observed in skin scrapings or biopsies, but the skin disease responded very well to treatment with selamectin. The four other cats of the household remained healthy throughout the episode. Multiple skin scrapings were taken from them, but no mites were detected. Nevertheless, all of the cats were treated with selamectin. The owner suspected that the source of the *D. gatoi *infestation was a cat show.

Household 3 consisted of two cats; one Exotic cat (case 3A) and one Persian cat (case 3B). The disease started with an intense pruritus of several months in duration in cat 3A. A few months later, cat 3B started to scratch as well. Prior to being admitted to the feline practice in February 2004, both cats had been treated twice with ivermectin and several times with corticosteroids and fed an elimination diet to exclude adverse food reactions without relief. Multiple skin scrapings from both cats revealed demodectic mites morphologically typical of *D. gatoi*. The cats were treated with repeated ivermectin injections followed by repeated treatments with selamectin (Table 2). As there were no signs of improvement within three months, the treatment was switched to bathing with 0.0125% amitraz solution (Table 2). Four weeks later, the cats were less pruritic and fur regrowth had appeared on the alopecic sites. The cats continued to lick their paws. As one dead mite was found in the skin scrapings from cat 3B, the amitraz bathing was continued for an additional three weeks. The cats remained healthy during the subsequent two-year follow-up. The owner suspected that the cats had contracted the *Demodex *infestation from a cat show in the Czech Republic.

Household 4 consisted of two Cornish Rex cats. The younger cat (Case 4A) was imported from Sweden. It was licking itself and some pruritic papules were noticed on the skin already upon arrival in Finland in February 2005. The cat developed a severe pruritic dermatosis within the following months. The dermatosis was characterized by scaling, alopecia and severe self-inflicted excoriations (Figure 1). Numerous mites were observed on tape strips. Amitraz bathing with 0.0125% solution was initiated (Table 2). The pruritus diminished already after the first dip. After the second dip, the cat was symptomless and regrowth of fur was observed within a few weeks. Despite a lack of symptoms, the other cat of the household was treated as well.

Household 5 consisted of a young Siamese cat (Case 5A) and an older Burmese cat (Case 5B). Both cats suffered from an intense pruritus, licking and scratching associated with secondary lesions for several months. Prior to the diagnosis, both cats had been treated once with selamectin and later with an imidacloprid-moxidectin compound (Table 2). In November 2005, several skin scrapings, tape strips and a fungal culture had been negative. Skin biopsies from both cats were submitted to a histopathological laboratory (Patovet, Helsinki, Finland). In all biopsies, mild hyperplastic hyperkeratotic perivascular dermatitis was present, and a few *D. gatoi *mites were observed within the keratin layer of one biopsy from cat 5B. The medication was continued by giving 1 mg ivermectin perorally every other day for 10 weeks. The pruritus disappeared within a few weeks and regrowth of fur was observed on the alopecic sites. Five months later, no mites were detected in the skin scrapings, tape strips or skin biopsies, and both cats were clinically healthy. The owner suspected that cat 5A had brought the mite infestation from its birth cattery.

Case 6A was a 10-month-old Cornish Rex cat living in a household of six cats. The cat was clinically healthy, but in July 2006 it was presented to a veterinarian for skin scrapings as a precaution in accordance with the breeder's recommendation. The cat was non-pruritic and no findings or clinical signs suggestive of a *D. gatoi *infestation were observed by the veterinarian. However, the skin scrapings revealed several *D. gatoi *mites representing different developmental stages, from egg to adult mites. All cats in the household were bathed with lime sulphur once a week (Table 2). After three bathings, new skin scrapings were taken and a few mites were still observed. The infested cat was bathed six times and the other cats four times. All cats, including the young one with the confirmed infestation, remained symptomless throughout the course of the events.

## Discussion

In the literature, feline demodecosis is presented as a skin disease caused by *D. cati*, *D. gatoi *or a third, yet unnamed *Demodex *species [2-4,6]. Although they belong to the same *Demodex *genus, the clinical picture of the disease differs according to the *Demodex *species involved. It is presumed that transfer of *D. cati *- and the same applies to most *Demodex *spp. also in other animals - occurs primarily between a mother and offspring during nursing [10]. In most circumstances, *D. cati *is considered a harmless resident of a cat's hair follicles [3,10]. When a generalized demodecosis is caused by *D. cati*, it typically manifests in the periocular skin, eyelids or external ear canal and is associated with an underlying immunological disorder or systemic disease [3,10,11]. By contrast, *D. gatoi *is able to cause a generalized, extremely pruritic skin disease in otherwise healthy cats of all ages [2-4,12-15]. Here, cats of different ages from several households had a clinically apparent *D. gatoi *infestation.

The stubby and roundish appearance of the mites was regarded as a morphological feature typical of *D. gatoi *in all cases presented here. The skin scrapings taken from a symptomless cat (6A) yielded the largest amount of *D. gatoi *mites, representing all developmental stages (Figure 2). Morphology and measurements of male (length about 90 μm), female (length about 110 μm), ovoid egg (40 μm × 25 μm) and larval and nymphal stages were in accordance with the descriptions of Desch and Steward [16]. *D. gatoi *can easily be differentiated from *D. cati*, which is slender and approximately twice as long [3,16]. Among the demodectic mites identified to date, *D. gatoi *most strongly resembles *D. criceti*, the demodectic mite of a hamster. However, despite the marked resemblance and similar size, some obvious morphological differences that can be identified by an entomologist exist [16].

The niche of *D. gatoi *in the superficial keratin layer of the skin and its role as a contagious, primarily pathogenic parasite are features that are atypical of demodectic mites. These features and *D. gatoi *not being identified earlier than 1981 (*D. cati *was found over a century earlier, in 1877) raise some questions. Although demodectic mites are regarded as host-specific, one explanation might be that *D. gatoi *was originally a commensal of some other mammalian species and has only recently been introduced to cats. Interestingly, a similar short-bodied demodectic mite has been described in a dog [17-19].

Based on the observations of the cases reported here, the infestation can be either symptomless or intensively pruritic. Secondary lesions, associated with barbering, and scratching were common. Broken and stubbled hairs, alopecia and mild to moderate scaling were observed in all pruritic cases. Also self-inflicted excoriations and wounds were often seen on the skin (Figure 1). Unexpectedly, the cat carrying the heaviest mite burden showed no signs. This suggests that the pathogenesis of the clinical disease may be based on a hypersensitivity reaction. If so, the cats that are not sensitized may remain healthy, although they can host an enormous amount of mites that can readily be transferred to other cats during contact. As some of the cats in the same household showed no symptoms and were presumably free of mites, it is also possible that some cats may possess an unsuitable cutaneous microenvironment (for mites) or may be able to spontaneously clear the infestation either by grooming or through their immune responses.

All of the cases reported here were pedigree cats (although these represent a minority of the Finnish cat population), and several of them had been in cats shows. To date, no reports of this mite occurring in domestic short-haired cats in Finland exist. This suggests that some purebred cats may be more susceptible to (clinically apparent) *D. gatoi *mite infestation or a relatively new feline disease that has started to spread through breeding catteries and cat shows has emerged. Show cats are not usually allowed to roam free, but national and international cat shows, multi-cat households, international cat trading and cat breeding enable contact between cats, and they may all be factors contributing to the transmission of *D. gatoi *infestations. The owners of the cats presented here were convinced that the source of *D. gatoi *infestations was either a cat show or a breeding cattery, but further studies are warranted to evaluate the role of cat shows and catteries in the epidemiology of *D. gatoi *infestations.

In four households, the diagnosis was obtained through skin scrapings or tape strips. These methods are easy, fast and reliable in the diagnosis of *D. gatoi *infestation when mites are detected. However, mites were not found in all pruritic cats. In two of the households, skin biopsies were needed to confirm infestation. The difficulties in demonstrating the mites have been highlighted by several authors [2-4,6,15]. Some sensitized cats may develop severe pruritus in the presence of only a few mites. In addition, intensive self-grooming by pruritic cats may considerably reduce the *D. gatoi *burden in the keratin layer of the skin, making detection of mites even more challenging. Due to their small size and translucency, *D. gatoi *mites may also go unnoticed under the microscope [2-4,6,15]. The latter problem can often be mitigated if the condenser of the microscope is lowered to reduce the amount of light and the diaphragm is narrowed to increase the contrast.

Skin biopsies were obtained for six cats and in two households the diagnosis was based on histopathological findings. A definitive diagnosis can be obtained through histopathology when stubby *Demodex *mites are present within the superficial keratin layer of the skin. As in skin scrapings, if the cat is extremely pruritic and constantly overgrooming, the mites may be absent in a biopsy as well. In the cases presented here, numerous mites were observed within the superficial keratin layer in two cases (Figure 3a), few mites in two cases and no mites in two cases. In all biopsies, mild acanthosis, mild lamellar hyperkeratosis and mild nonsuppurative perivascular dermatitis were present. In one sample, lymphocytic exocytosis was also observed (Figure 3c). These lesions without a finding of mites should be regarded as non-specific and suggestive of a hypersensitivity reaction (e.g. allergic dermatitis). Epidermal erosion and serocellular crusting were present in two cases as typical secondary lesions associated with self-trauma. The histopathology observed in these cases is in accordance with published descriptions [5].

Dermatophytosis, allergies, other ectoparasites, especially *Notoedres cati *and *Cheyletiella*, and feline psychogenic alopecia are the major differential diagnoses for *D. gatoi-*associated dermatitis [2-4,6,14,15]. Intense self-grooming and hair plucking may result in a bilateral symmetrical alopecia, hindering the diagnosis [2-4,6,14,15]. A pruritic skin disease affecting several cats in the same household should alert the practitioner to the possibility of a contagious aetiology.

Considering the diagnostic difficulties, some authors suggest that *D. gatoi *dermatitis should be treated whenever suspected [2-4,6]. However, all currently used treatment protocols are arduous and time-consuming, and therefore not ideal in therapeutic trials. Without a doubt, an obvious need exists for a reliable diagnostic test for *D. gatoi*-associated skin disease and *D. gatoi *infestation. Such a test would enable more information about the actual prevalence and virulence of *D. gatoi *to be obtained.

According to the literature, the treatment of choice for feline *D. gatoi *demodecosis is a weekly 2% lime sulphur dip [2-4,6]. The superficial location of the mites is believed to account for the favourable response to lime sulphur [4]. However, because of its foul odour and tendency to stain light-coloured coats, some owners and practitioners are reluctant to use it. Improvement should be observed after three or four dips, but usually the treatment protocol contains a minimum of 4-6 weekly dips [2-4,6,20]. The cats of one household here were treated with lime sulphur dips. The owner purchased the product from Sweden. However, as all cats remained symptomless, the clinical efficacy of the product could not be evaluated. The mites continued to be observed in skin scrapings after three dips, indicating that, despite the promising treatment results obtained in the USA, *D. gatoi *is quite tolerant to lime sulphur dips. Poor efficacy or even treatment failures with lime sulphur dips have been reported earlier [21].

As products containing lime sulphur were not available in Finland at the time of the study, other treatment options were considered. Nowadays, several endectocides are registered for cats. Imidacloprid-moxidectin was tried in one household, but it was ineffective against *D. gatoi *when used according to the manufacturer's instructions, despite a proven efficacy against several other ectoparasites. With selamectin, controversial results were obtained. In one household, favourable results were achieved, whereas treatment failures occurred in two other households. Increasing the dose or the frequency of treatments did not improve the efficacy. A similar finding was reported in an earlier study containing 17 cats with clinical demodecosis (14 with *D. gatoi*). Selamectin was ineffective when used weekly for six weeks at a dose of up to 20 mg/kg bw, although clinical improvement was noted in 14 of the 17 cats [22]. In our study, repeated treatments with ivermectin injections did not cure the disease. However, the cats in one household were successfully treated by giving them 1 mg ivermectin perorally every other day for 10 weeks, indicating that macrocyclic lactones actually may be effective in the treatment of *D. gatoi *infestation. However, the optimal dose and treatment intervals remain obscure.

Amitraz, although not licensed for feline use, has been successfully used in the treatment of feline demodecosis, also in cases associated with *D. gatoi *[20,22]. It is usually used at a 0.0125 - 0.025% concentration. Anorexia, depression and diarrhoea are the most common side-effects [20,22]. In our study, amitraz was successfully used in three households. However, one of the owners noted some depression in cats.

To avoid spreading the infestations, it is important to determine, how long after the first treatment the infested cats or their housemates remain infective. Affected cats should also be denied contact with other cats. The organizers and exhibitors of cat shows pay much attention to prevention of infectious diseases, particularly dermatophytoses. *D. gatoi *- as an emerging contagious skin disease in cats in Finland - certainly belongs to the infectious agents that should be under close observation.

*D. gatoi*-associated dermatitis resembles in many respects the canine scabies caused by *Sarcoptes scabiei*. Both mites dwell in the superficial keratin layer of their host's skin. Both are contagious and often cause an intensively pruritic dermatosis. Some individuals remain symptomless despite infestation. Both of the mite species may be difficult to demonstrate on tape strips, in skin scrapings or biopsies. However, one major difference exists: while canine scabies can easily be controlled with the aid of modern companion animal avermectins or milbemycins, a convenient way to treat *D. gatoi*-associated dermatosis and an optimal duration of treatment remain to be determined.

## Authors' note

As further evidence for the emergence of *D. gatoi *in Finland, the authors (SS, LSK, KJ and RLS) - since writing this paper - have diagnosed additional cases (data not shown) of *D. gatoi*-associated pruritic skin disease in several pedigree cats (e.g. in Russian Blue cats).

## Competing interests

The authors declare no competing interests. SS is currently employed by Pfizer Animal Health.

## Authors' contributions

SS was responsible for the histopathological examinations, taking the micrographs, the morphological description and the scanning electron microscopy of the parasite. LSK, KJ, JK, KV and RLR were responsible for the clinical examinations and monitoring the efficacy of the treatment options. They were also involved in collecting the data and samples from the clinical cases. SS and LSK drafted the manuscript. All authors approved the final manuscript.
